# *Paramecium tetraurelia* basal body structure

**DOI:** 10.1186/s13630-016-0026-4

**Published:** 2016-02-08

**Authors:** Anne-Marie Tassin, Michel Lemullois, Anne Aubusson-Fleury

**Affiliations:** Institute for Integrative Biology of the Cell (I2BC), CEA, CNRS, Univ. Paris Sud, Université Paris-Saclay, 1 Avenue de la Terrasse, 91198 Gif sur Yvette, France

**Keywords:** *Paramecium*, Basal body assembly, Basal body docking, Transition zone, Ciliogenesis

## Abstract

*Paramecium* is a free-living unicellular organism, easy to cultivate, featuring ca. 4000 motile cilia emanating from longitudinal rows of basal bodies anchored in the plasma membrane. The basal body circumferential polarity is marked by the asymmetrical organization of its associated appendages. The complex basal body plus its associated rootlets forms the kinetid. Kinetids are precisely oriented within a row in correlation with the cell polarity. Basal bodies also display a proximo-distal polarity with microtubule triplets at their proximal ends, surrounding a permanent cartwheel, and microtubule doublets at the transition zone located between the basal body and the cilium. Basal bodies remain anchored at the cell surface during the whole cell cycle. On the opposite to metazoan, there is no centriolar stage and new basal bodies develop anteriorly and at right angle from the base of the docked ones. Ciliogenesis follows a specific temporal pattern during the cell cycle and both unciliated and ciliated docked basal bodies can be observed in the same cell. The transition zone is particularly well organized with three distinct plates and a maturation of its structure is observed during the growth of the cilium. Transcriptomic and proteomic analyses have been performed in different organisms including *Paramecium* to understand the ciliogenesis process. The data have incremented a multi-organism database, dedicated to proteins involved in the biogenesis, composition and function of centrosomes, basal bodies or cilia. Thanks to its thousands of basal bodies and the well-known choreography of their duplication during the cell cycle, *Paramecium* has allowed pioneer studies focusing on the structural and functional processes underlying basal body duplication. Proteins involved in basal body anchoring are sequentially recruited to assemble the transition zone thus indicating that the anchoring process parallels the structural differentiation of the transition zone. This feature offers an opportunity to dissect spatio-temporally the mechanisms involved in the basal body anchoring process and transition zone formation.

## The organism

**Fig. 1:**
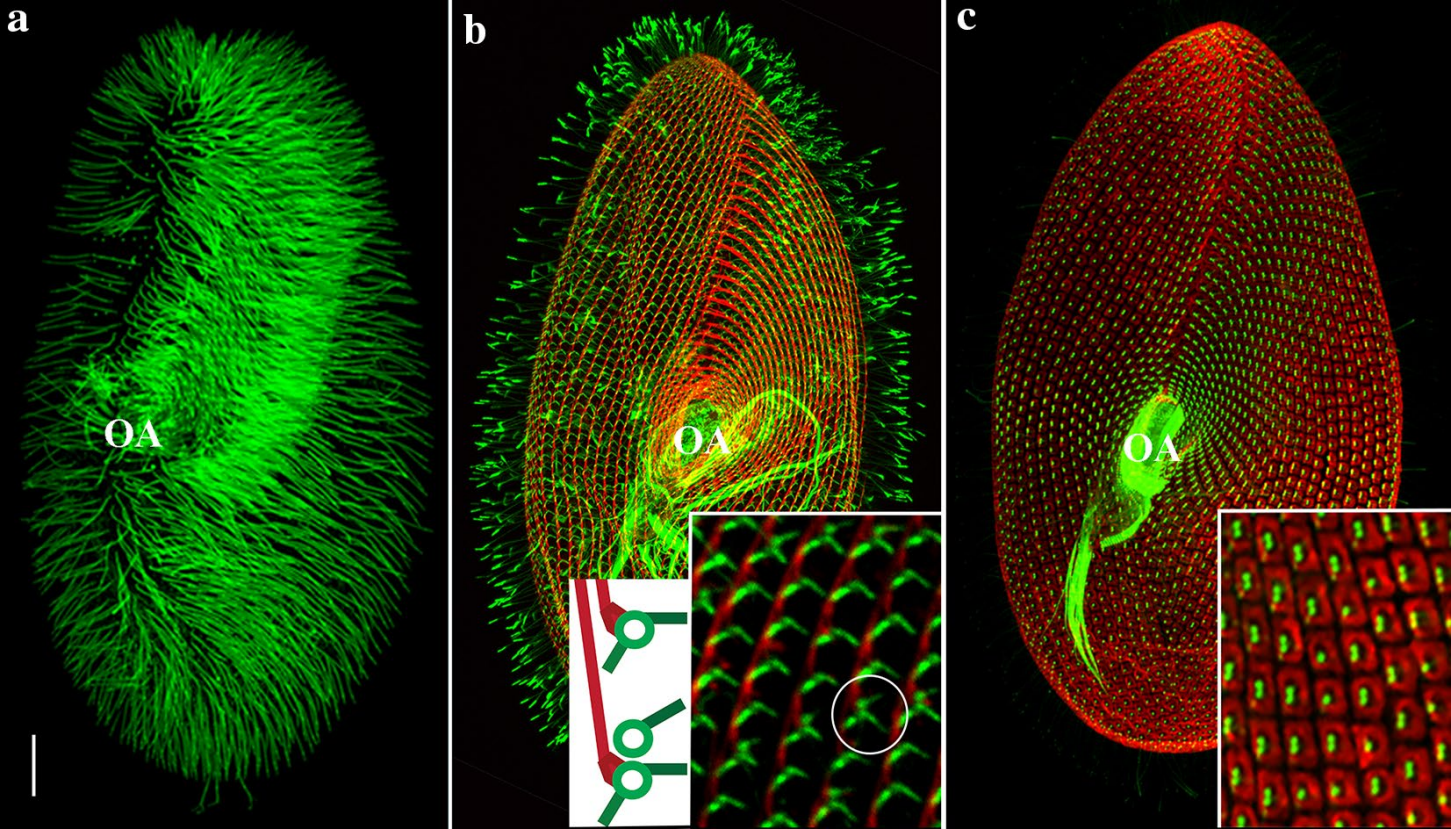
Pattern of cilia and basal bodies in *Paramecium*. Images are projections of confocal images taken at the level of the ventral side of the cell. For details in immunofluorescence procedures, see [1]. *Bars* 20 μm. *Insets*:  ×5. **a** Ciliary pattern. The cell is labelled with an antibody directed against monoglycylated tubulin. The anterior left half-quarter appears brighter because it is more densely ciliated than other parts of the cell. Beating of these cilia guides the water current towards the cell centre where the oral apparatus (OA) is located. At the posterior pole of the cell are few longer non-motile cilia. **b** Pattern of the basal body-associated rootlets. *Green*: microtubular rootlets, decorated with an anti-acetylated tubulin; *red*: striated rootlets, decorated with an antibody specific for striatins [2]. Striated rootlets of successive basal bodies cluster to form a continuous bundle along the right of the basal body row. Cartoon: one (*top*) or two (*bottom*) transverse microtubular ribbons are detected in association with single or paired basal bodies, respectively. *Circle:* paired basal bodies with two transverse ribbons. *OA* oral apparatus. **c** Pattern of cortical units. *Red*: epiplasm units, decorated with an antibody specific for epiplasmins [3]; *green*: basal bodies labelled with an anti-polyglutamylated tubulin [4]. *OA* oral apparatus

*Paramecium tetraurelia* is a unicellular eukaryote belonging to the Chromalveolata kingdom, Ciliophora phylum. It is a free-living bacteriophagous organism that is easy to cultivate, usually found in freshwater where it can swim and capture its preys thanks to its ca. 4000 cilia (Fig. 1).

## Basic basal body structure

In *Paramecium,* basal bodies are arranged into parallel rows, the kineties, patterned along the antero-posterior axis of the cell, and their rotational polarity is marked by the asymmetrical organization of their associated structures (Fig. 1b). These basal bodies are anchored at the cell surface and embedded in a superficial cytoskeletal layer, the epiplasm, which partitions the cell surface in cortical units [5–8]. In interphasic cells, either one or two basal bodies are anchored in the middle of each cortical unit (Fig. 1c). Basal body duplication occurs close to the mother basal body, which, contrary to mammalian cells, is permanently anchored at the cell surface. During cell division, several successive duplication waves of basal bodies and associated appendages together with the formation of new cortical units lead to the duplication of the whole cell pattern [7].

**Fig. 2:**
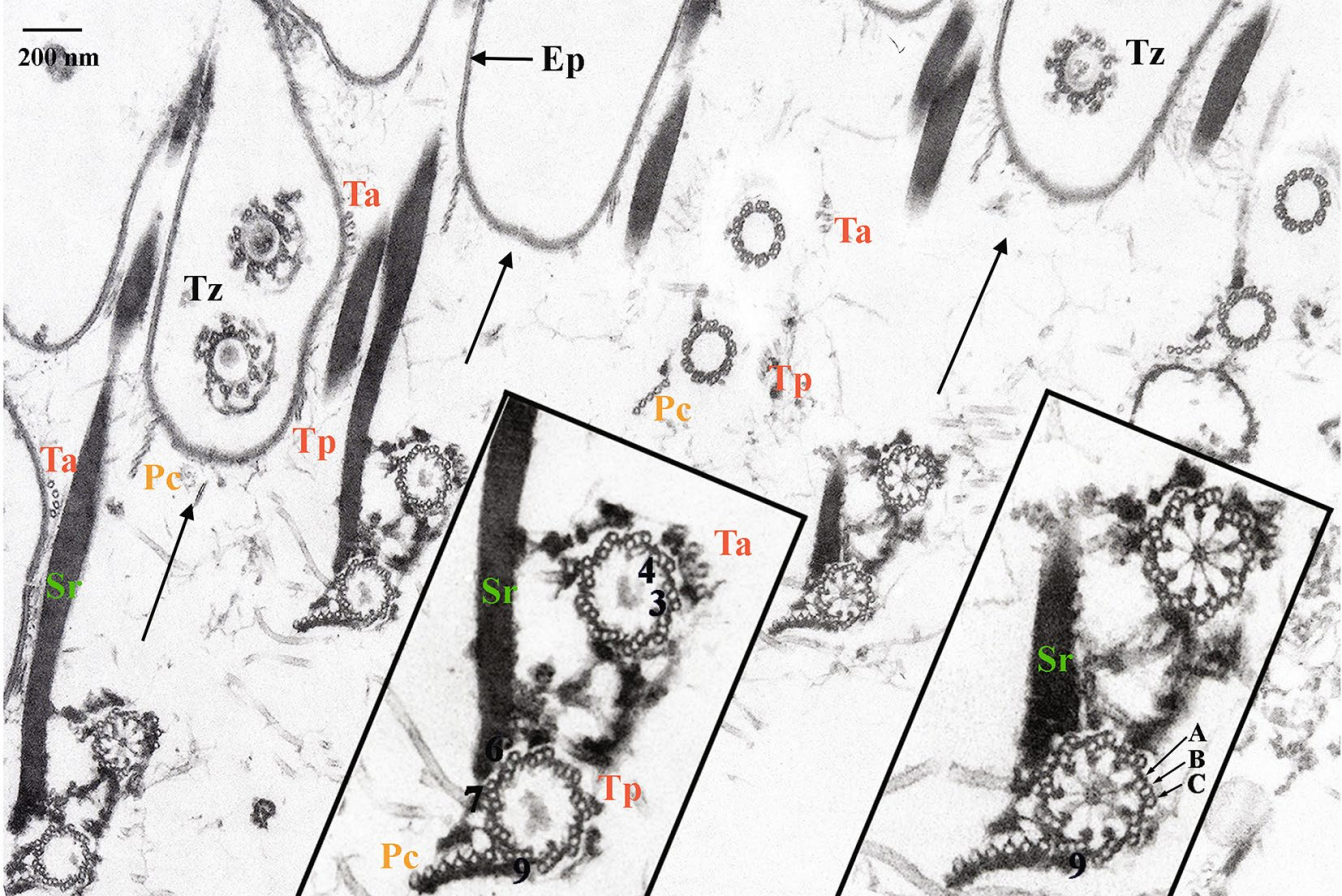
Organization of the cell surface at the ultrastructural level; tangential section of a detergent-extracted *Paramecium* cell fixed in the presence of tannic acid (modified from 12). *Bar* 200 μm. *Insets*: ×2. The *arrows* point towards the anterior of the basal body rows. Basal bodies are transversally cut at the level of the cartwheel (*right inset*) or at the level of the transition zone (Tz) (*left inset*). At their base, paired basal bodies are connected together by a complex set of links (*insets*). The post-ciliary rootlet (Pc) originates close to the triplet 9 (according to the Grain’s triplet numbering in Ciliata [25]), the transverse anterior (Ta) and posterior (Tp) rootlets close to the triplets 3 and 4, and the striated rootlet (Sr) is connected to the triplets 6 and 7. These three rootlets, associated with each basal body pairs, extend from the basal body bases towards the cell surface where they connect the epiplasm (Ep). The anterior basal body is connected in its proximal part to the striated rootlet (*insets*). At the proximal level, the Pc rootlet is connected to the ciliary rootlet by a set of links (*insets*). At the Tz level, links are detected in association with each microtubule doublets. Tubules A, B and C composing the basal body wall are indicated on the *right inset*

**Fig. 3:**
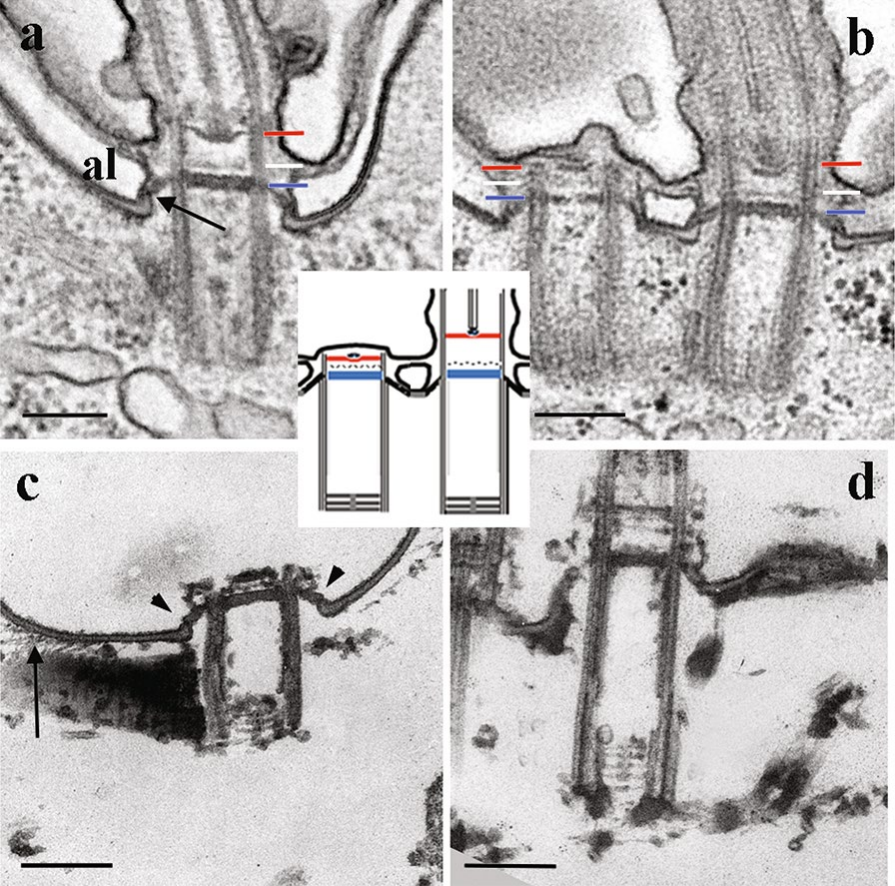
Longitudinal sections through *Paramecium* basal bodies after glutaraldehyde/osmium classical fixation (**a**, **b**) or with an additional tannic acid treatment performed after cell permeabilization (**c**, **d**). *Bars* 200 nm. **a** Connection between the Tz and the cell surface. Inside the basal body, the Tz is organized in three plates: the terminal plate (*blue line*), the intermediate plate (*white*) and the axosomal plate (*red line*). Outside the basal body, the terminal plate extends to link the epiplasm (*arrow*). Inside the basal body, dense granules are observed. *Al* alveolar sac , a vacuolar system located beneath the outer cell membrane found in all representatives of the Chromalveolata. **b** Comparison of Tz of non-ciliated and ciliated basal bodies: Tz of ciliated basal bodies is more extended than that of non-ciliated basal bodies, but the three plates and the connection with the epiplasm are detected in both of them. **c**, **d** (modified from [12]): short (**c**) and long (**d**) basal bodies. The cartwheel is longer in the long basal body. The three plates, as well as the connection with the epiplasm (*arrowheads*) can be observed on the short non-ciliated basal body; connections between the striated rootlet and the epiplasm appear as delicate links (*arrow*). A schematic representation of anchored ciliated and non-ciliated basal bodies has been inserted in this figure showing the transition zone with its three plates: the terminal plate (*blue*), the intermediate plate (*discontinuous line*) and axosomal plate (*red*)

*Paramecium* basal bodies display a ninefold symmetry of microtubule blades with, at their proximal end, microtubule triplets surrounding a cartwheel that remains present all along their life cycle [9, 10] (Fig. 2). Microtubule triplets (A, B and C tubules) end up at the level of the transition zone and microtubule doublets are observed above [11] (Fig. 2). All *Paramecium* basal bodies show a similar organization, but their length varies between 330 and 600 nm depending on their location on the cell cortex (Fig. 3). The height of the cartwheel correlates with the basal body length; the longest are present in the oral apparatus, while the shortest are scarce and found only in the cell cortex [12].

Dute and Kung [11] have studied the structure of the *Paramecium* transition zone in detail using both thin-section and freeze-fracture electron microscopy. This transition zone is particularly well delimited and organized in three distinct plates namely the terminal, the intermediate and the axosomal plates (Fig. 3). The terminal plate marks the boundary between the basal body and the transition zone. Hufnagel [5] showed that this plate is organized around a central rim, from which nine spokes radiate into the gap between the microtubule doublets. This plate is at least partially built up with epiplasmins, the protein components of the epiplasm [8]. Nine perforations in the epiplasm ring located around the microtubule doublets have been observed in both *Paramecium* [5] and *Tetrahymena* [13]. These structures correspond to the ciliary pores described by Ounjai et al. [14] in *Tetrahymena* basal bodies after potassium phosphotungstate treatment to remove the microtubules. Transitional fibres are difficult to observe but a pinwheel structure surrounding the microtubule doublets has been disclosed. It originates from the terminal plate and ends beneath the plasma membrane. Close to the axonemal plates, peg-like Y-shaped structures called Y-links project from the common wall of the A and B tubules [11] (Fig. 2). In *Paramecium*, docked basal bodies are not systematically ciliated so that both non-ciliated and ciliated docked basal bodies can be observed in the same cell. Growth of cilia is accompanied by modifications of the structure of the transition zone which appears more collapsed in anchored non-ciliated basal bodies than in the ciliated ones (Fig. 3) [15]. Two recent papers concerning the function of two transition zone proteins MKS1 and MKS3 [16, 17] report loss of cilia after their depletion suggesting that in *Paramecium* MKS1 and MKS3 are essential for cilium stability as in mammals [18].

In the tubulin superfamily comprising six members, δ-tubulin has been shown to be required for the C-tubule assembly [19], while ε-tubulin was necessary for B- and C-tubule assembly or stabilization of the microtubule triplet. In addition, basal body duplication is impaired after ε-tubulin depletion [20]. A mutation found in *sm19*, encoding the rare eta now called ζ [21], was reported to inhibit basal body duplication and to induce the delocalization of γ-tubulin [22, 23].

## Additional basal body structures or accessory structures

Three major appendages typical of all Ciliata are found to be associated with *Paramecium* basal bodies: a long striated rootlet crossing over several cortical units and two microtubular rootlets, the transverse microtubules and the post-ciliary microtubules [12, 24, 25]. They protrude asymmetrically from the proximal part of the basal bodies. The direction of these cytoskeletal appendages correlates with the antero-posterior axis of the ciliary row; the pattern of these ciliary rows correlates with the global cell polarity with its right–left asymmetry and antero-posterior axis (Fig. 1b). The striated rootlet (kinetodesmal fibre) is connected to the right side of the basal body and extends along the basal body row towards the anterior pole of the cell (Fig. 1b). The transverse microtubules originate close to the basal body in its left anterior quarter, and run perpendicular to the basal body row towards the left side of the cortical unit. The post-ciliary microtubules originate close to the basal body in its posterior right quarter and extend towards the posterior pole of the basal body row [12, 24, 25]. The striated rootlet guides the new basal body during its positioning [10].

Lynn [26] has proposed that the basal body and its associated set of rootlets form the kinetid. Monokinetids (single basal bodies) display the three rootlets, while in dikinetids (paired basal bodies) only one post-ciliary and one striated rootlet are present. They are associated with the posterior basal body, while both anterior and posterior basal bodies have associated transverse microtubular rootlets (Fig. 2). In both mono- and dikinetids, a delicate system of fibres links the basal bodies to their rootlets [12]. Further details on the fate of permanent appendages during duplication are available in [10].

An additional transient appendage, called the anterior left filament (ALF), develops at the anterior left from the mother basal body (triplets n°4) before duplication and disappears once the new basal body is docked at the surface [27]. This transient filament requires Centrin3 for its formation and is assumed to be involved in the tilting up of the new basal body allowing its anchoring [27].

## Basal body origins

All basal bodies develop from a pre-existing mother basal body and in contrast to other ciliates such as *Oxytricha* [28, 29], *Sterkiella* [30] or *Paraurostyla* [31], no de novo assembly of basal bodies has ever been observed in *Paramecium*.

## Basal body life cycle and other functions

In *Paramecium*, basal bodies are dedicated to organize cilia required for motility, food uptake, sensory functions and cell–cell recognition during sexual reproduction. Basal bodies never act as a centrosome to organize the mitotic nuclear spindle. They duplicate close to their parents and remain anchored at the cell membrane during the whole cell cycle. During division, new basal bodies act as organizing centres for the assembly of new cortical units. Their microtubular rootlets probably act as templates for the assembly of a superficial sub-membranous spindle, the cytospindle transitory detected during division [32, 33].

## Identification of basal body components

In this species, no proteomic or genomic screens dedicated to dissecting basal body composition have been performed so far. However, a proteomic screen of isolated cilia [34] and a study of transcriptomic changes during ciliary biogenesis in response to deciliation have been carried out to understand the ciliogenesis process [35]. In addition, a ciliary membrane proteomic analysis has been reported recently [36]. Results from these analyses were all included in the Cildb database (http://cildb.cgm.cnrs-gif.fr/) [34, 37]. Together with 66 high-throughput studies from 15 eukaryotes having centrioles/basal bodies and cilium, they enabled Carvalho-Santos [38] to establish simplified phylogenetic profiles of the structure and function of these organelles.

## Notable basal body findings

In 1965, Beisson and Sonneborn [39] demonstrated that the polarization of newly assembled kinetids is determined by the polarity of the cortical environment existing at the time of their development, leading to the concept of “cortical inheritance” or structural memory.

In 1968, Dippell [9], thanks to thousands of basal bodies arising almost synchronously in known positions at a recognizable stage, has followed the duplication steps of the basal body and dissected the formation of the microtubule wall in a pioneer electron microscopy study.

In 1994, Redecker et al. [40] have discovered a new tubulin modification using *Paramecium* axonemal tubulin. This post-translational modification, which affects kinetid microtubules a lag-time after their assembly, has been suggested to act as a marker to discriminate parental and new structures during cell division [33].

Ruiz et al. [23], taking advantage of the fact that cells continue dividing after inhibition of basal body duplication, demonstrated for the first time the requirement of γ-tubulin for basal body duplication. They also demonstrated first that Centrins are required for basal body positioning but not for its duplication per se [41].

In 2000, a novel member of the tubulin superfamily was identified using the sm19 mutant, and called η/ζ-tubulin. The mutations in the gene encoding this protein caused an inhibition of basal body duplication [42]. Further physiological and genetical studies indicate an interaction with microtubules [22]. In a recent functional study of ζ-tubulin in *Xenopus* [21], no evident basal body defect was observed, leading to the conclusion that ζ-tubulin is involved in basal body orientation and distribution and might function by interacting with other tubulins.

Finally, studying the process of basal body anchoring through a combination of GFP-fusion protein expression, RNAi and low-resolution electron microscopy, Aubusson-Fleury et al. [15] discovered that three proteins, Centrin2, FOR20 and Centrin3, are sequentially recruited to allow basal body assembly and anchoring. Interestingly, unanchored basal bodies are arrested at a precise step in their transition zone assembly: Centrin2 or FOR20 depletion leads to an almost absence of transition zone. By contrast, Centrin3-depleted cells exhibit unanchored basal bodies with a fully assembled transition zone, similar to that of anchored unciliated basal bodies. These results strongly suggest that the sequential recruitment of these proteins parallels the transition zone assembly process.

## Strengths and future of basal body research in *Paramecium*

The strengths of the *Paramecium* model rest in its long established status as a genetic model [43] and its large number of basal bodies. Their regular arrangement over the cell surface, as well as the precise choreography of their duplication, facilitates functional analyses of ciliary proteins both at the cellular and ultrastructural levels. Such a precise organization has allowed, in the past, to perform pioneer and high-quality ultrastructural studies of the basal body duplication process [9] and the transition zone arrangement [11]. The coupling of rapid and non-costly functional analyses of candidate genes based on RNAi knock-down using feeding techniques [44], and overexpression of tagged-fusion proteins by nuclear microinjection of DNA, makes *Paramecium* an outstanding and efficient model to study basal body assembly and functions. It is also particularly suitable for assessing the process of basal body anchoring, since anchoring defects are detected by immunofluorescence using specific antibodies and can be easily followed from the early steps of protein depletion.

## What are the prospects for future work?

Unravelling the structural aspects of cilia assembly is fundamental in the study of ciliopathies. Owing to the motility of its cilia, *Paramecium* is obviously an appropriate model to study the function of proteins involved in primary ciliary dyskinesia (PCD), a pulmonary disease arising from immotile respiratory cilia. The transition zone acts as a filter between the cellular and the cilium compartments and houses many proteins involved in human ciliopathies. However, the structural bases of the ciliary gate function are currently unknown. The large number of basal bodies in *Paramecium,* and the easiness to prepare *Paramecium* cell cortices, will allow studying the structural organization of the *Paramecium* transition zone using high-resolution microscopy such as cryo-electron tomography [45] or scanning transmission electron tomography. In addition, in *Paramecium,* the basal body docking is not systematically coupled to ciliogenesis, which occurs throughout the cell cycle (Aubusson-Fleury, in preparation). Therefore, both non-ciliated and ciliated docked basal bodies can be observed in the same cell. In anchored non-ciliated basal bodies, the transition zone appears more collapsed than in the ciliated ones, suggesting that maturation of this region occurs during axoneme extension [15]. Multi-disciplinary approaches, combining biochemical, molecular and cell biology techniques with high-resolution ultrastructural approaches, will permit characterization of the molecular and structural bases of the transition zone assembly process in a 4D-space. This will open the way to further studies linking atomic structure with transition zone assembly.

## Abbreviations

Tz: transition zone
Pc: post-ciliary rootlet
Ta: transverse anterior
Tp: transverse posterior
Sr: striated rootlet
Ep: epiplasm
OA: oral apparatus
Al: alveolar sac
